# The mesopic negative response (MeNR): a novel approach to assess retinal ganglion cell function within the rod pathway

**DOI:** 10.1007/s10633-025-10040-3

**Published:** 2025-07-09

**Authors:** J. Jason McAnany, Jason C. Park, Pablo Barrionuevo, Dhara Shah, Thasarat Sutabutr Vajaranant, Ahmad A. Aref, Deepak P. Edward, Robert A. Hyde

**Affiliations:** 1 Department of Ophthalmology and Visual Sciences, University of Illinois at Chicago, 1855 W. Taylor St., MC/648, Chicago, IL 60612 USA; 2 Department of Bioengineering, University of Illinois at Chicago, 851 South Morgan St., Chicago, IL 60607 USA; 3 Philipps Universität Marburg, FB04 Psychologie, Gutenbergstraße 18, 35032 Marburg, Germany; 4 Instituto de Investigación en Luz, Ambiente y Visión, CONICET - UNT, Av. Independencia 1800, T4002BLR, San Miguel de Tucumán, Tucumán Argentina; 5 King Khaled Eye Specialist Hospital and Research Center, Riyadh, Kingdom of Saudi Arabia

**Keywords:** Electroretinogram, Silent substitution, Photoreceptor-directed ERG, Glaucoma, Mesopic negative response, Photopic negative response

## Abstract

**Purpose:**

The photopic negative response (PhNR) and pattern ERG, both established electrophysiological measures of retinal ganglion cell (RGC) function, are recorded under photopic conditions. The purpose of this study was to describe the mesopic negative response (MeNR), a novel marker of RGC function within the rod pathway.

**Methods:**

Ten visually-normal controls (mean age ± SD 54.6 ± 5.6 years) and 12 patients with severe primary open-angle glaucoma (mean age ± SD 58.4 ± 4.9 years) participated. Light-adapted, full-field ERGs were elicited by a rod-isolating pulse generated on the principle of silent substitution (0.46 scot. cd/m^2^; 55% contrast; 40 ms) presented against a steady background (0.30 scot. cd/m^2^). In addition, (1) the PhNR was recorded; (2) subjects were dark-adapted for 20 min and the ISCEV DA 0.01 was recorded.

**Results:**

The normal rod-isolated pulse response was characterized by a positive potential at 85 ms followed by a slow negative potential (MeNR) at 175 ms following the pulse. The mean (± SEM) amplitude of the positive potential was similar for the control (13.4 ± 1.2 µV) and glaucoma (11.6 ± 1.35 µV) groups (*p* = 0.33), and was correlated with the DA 0.01 amplitude (r = 0.71, *p* < 0.001). The amplitude of the MeNR was significantly (*p* < 0.001) attenuated for the glaucoma group (4.5 ± 0.7 µV) compared to the controls (9.7 ± 1.25 µV), and was correlated with the PhNR amplitude (r = 0.86, *p* < 0.001).

**Conclusions:**

Rod-isolated ERGs can be obtained without dark-adaptation using silent-substitution. The positive potential and MeNR of the rod-isolated response appear to be generated by rod bipolar cells and RGCs, respectively. In severe glaucoma, the positive (bipolar cell) potential was not significantly affected, whereas the MeNR was significantly reduced. MeNR analysis may be useful for studying RGC function within the rod pathway.

**Supplementary Information:**

The online version contains supplementary material available at 10.1007/s10633-025-10040-3.

## Introduction

The photopic negative response (PhNR) [1], the scotopic threshold response (STR) [2], and the pattern electroretinogram (PERG) [3] are established approaches for non-invasive assessment of retinal ganglion cell (RGC) function. Used clinically, these tests have provided important information regarding RGC function in patients with ocular hypertension [4], glaucoma [5], and other optic neuropathies [6]. The PhNR and PERG are performed under photopic conditions and reflect RGC function within the cone pathway [1, 3]. The scotopic threshold response (STR) of the full-field ERG provides an approach to assess RGC function within the rod pathway [2]. STRs are recorded in response to low luminance flashes, typically 500× to 10,000× lower than the ISCEV standard DA01 response, following careful dark adaptation [2, 7]. Given the low luminance stimulus requirement, partial light adaptation from stray room light can affect the STR, making recordings challenging in clinical environments [8]. There are relatively few reports of the STR in human subjects, although the response is more commonly measured in animal studies where laboratory illumination conditions are often better controlled and time constraints are less demanding [9].

The requirement for lengthy dark-adaptation is a burden in recording the STR and the ISCEV electroretinogram (ERG) series generally. One approach to overcome this limitation is to perform silent substitution ERG (also referred to as “photoreceptor directed” ERG), which permits rod pathway assessment without dark adaptation [10–12]. This approach is based on maintaining a constant quantal catch of the cone photoreceptors while simultaneously modulating the quantal catch of the rod photoreceptors. Under mesopic light levels, all four photoreceptor types (L cones, M cones, S cones, and rods) maintain sensitivity, requiring the use of a four primary system to achieve a constant cone quantal catch and “silence” the cones. As described elsewhere [13] and further below, this can be accomplished with commercially available ganzfeld stimulus sources that contain four independent primaries (e.g., red, amber, green, and blue LEDs).

This approach has been applied to generate rod-isolating flicker stimuli for recording ERGs from visually-normal individuals and patients with rod or cone dysfunction [12, 14–16]. For example, Maguire et al. [12] used slow (2 Hz) rod-isolating square-wave flicker presented to subjects who had prior adaptation to typical room illumination. This stimulus elicited a waveform with a fast positive component following stimulus onset (stimulus increment phase), a slow negative component while the stimulus was on (sustained stimulus increment phase), and a fast negative component following the stimulus offset (stimulus decrement phase). Responses were also obtained from individuals who had rod- or cone-pathway dysfunction to evaluate the origins of the rod-isolated ERG components. Patients with abnormal rod bipolar cell function lacked the fast positive and fast negative components but retained the slow negative component while the stimulus was on. By contrast, patients who had abnormal cone function with preserved rod function had responses similar to those of the visually-normal controls. Taken together, the results suggest that under the conditions tested: (1) the fast positive component of the rod-isolated response originates in rod ON bipolar cells; (2) the origin of the fast offset negative component is less clear, but may be generated by recovery of the rod ON bipolar cells. Although the origin of the slow negative component was not addressed, it appeared superficially similar to the PhNR, which is also a slow negative component that follows the b-wave (a fast positive potential). The temporally extended stimulus used by Maguire et al. [12] emphasized separate ON and OFF response components and it is unclear how the response morphology and underlying generators would change in response to short pulses of light that are more commonly used in clinical retinal electrophysiology.

The purpose of this study was to record rod-isolated ERGs elicited by short-duration (40 ms) pulses without dark adaptation. Although 40 ms is considerably longer than conventional luminance stimuli used for ERG recordings, it is shorter than the onset latency of the rod-isolated response (approximately 50 ms) reported by Maguire et al. [12]. We describe the morphology of the rod-isolated response and compare its amplitude characteristics to those obtained by conventional flash electrophysiology (ISCEV standard DA01 and PhNR measurements). To help infer the retinal source of the components, glaucoma patients with severe ganglion cell dysfunction were tested. We identified two components elicited by a brief rod-isolating pulse under mesopic conditions: a positive component that is likely rod ON bipolar cell driven (consistent with Maguire et al. [12]) and a slow negative component (termed the mesopic negative response; MeNR) that appears to be generated by RGCs within the rod-pathway.

## Methods

### Subjects

Ten visually-normal control subjects and 12 patients with severe primary open-angle glaucoma (POAG) participated. There was no significant difference between the age of the control group (mean age ± SD 54.6 ± 5.6 years) and the POAG group (mean age ± SD 58.4 ± 4.9 years). All subjects were recruited from the Department of Ophthalmology and Visual Sciences at the University of Illinois Chicago. Subjects who presented to the Glaucoma Service and had a diagnosis of severe POAG, based on the American Academy of Ophthalmology Preferred Practice Pattern [17], were recruited. The patients had definite retinal nerve fiber layer thinning on OCT (Cirrus 800, Carl Zeiss Meditec, Jena, Germany), with mean ± SEM RNFL thicknesses of 63 ± 7, 62 ± 4, 55 ± 6, and 50 ± 4 in the superior, inferior, nasal, and temporal quadrants, respectively. Visual field loss without secondary causes was apparent on standard automated perimetry (HVF 24–2 MD − 6.3 to − 33.5 dB). Visual acuity, which was not considered in the inclusion criteria, ranged from 20/20 to 20/60. Subjects with dense media opacities from whom HVF and/or OCT could not be perform were excluded. The studies were performed in accordance with the tenets of the Declaration of Helsinki, institutional review board approval was obtained at the University of Illinois Chicago, and the experiments were undertaken with the understanding and written consent of each subject.

### Rod-isolating stimuli

Details of the approach to achieve rod-isolated ERGs by silent substitution are provided elsewhere [12]. In brief, full-field pulse stimuli (40 ms in duration) were generated by and presented in a ColorDome desktop ganzfeld system (Diagnosys LLC, Lowell, MA), as described elsewhere [16, 18]. The subject adapted to a 0.5 phot. cd/m^2^ background that was generated by the sum of light emitted from red (0.28 phot. cd/m^2^), amber (0.04 phot. cd/m^2^), green (0.18 phot. cd/m^2^), and blue (0.005 phot. cd/m^2^) LEDs. During the 40 ms rod-isolating pulse presentation, the red and green LEDs were decremented in luminance (0.12 and 0.07 phot. cd/m^2^, respectively), the amber LED was incremented (0.31 phot. cd/m^2^), and the blue LED was essentially unchanged (0.005 phot. cd/m^2^). Given the spectral emission of the four LEDs and the human rod (V’[λ]) and cone (Smith–Pokorny, CIE1964 10^0^ Standard Observer) spectral sensitivities, this produced a 55% rod contrast pulse, whereas the cone contrast was effectively 0% (i.e., silent).

### Procedure and ERG recording

Prior to the monocular ERG recordings, the pupil of the tested eye was dilated with 1% tropicamide (Somerset Therapeutics, Hollywood, FL) and 2.5% phenylephrine hydrochloride drops (Lifestar Pharma, Mahwah, NJ), and the subject was exposed to typical room illumination. ERGs were recorded with DTL electrodes (Diagnosys, LLC); ear clip and gold cup (forehead) electrodes served as reference and ground, respectively. Responses were acquired with an Espion E3 electrophysiology console, with amplifier band-pass settings of 0.312–500 Hz at a sampling frequency of 1 kHz.

Prior to the rod-isolated ERG recordings, the subject was exposed to typical room illumination. During the recording, the subject was asked to fixate a dim marker within the ganzfeld bowl and 40 pulses were delivered at a rate of 2 Hz. The train of 40 pulses was repeated at least three times. Eye blinks and movements were omitted from the traces in post-processing analysis and a minimum of 33 responses was averaged for each subject. The rod-isolated response amplitude was measured from the baseline (0 µV) to the first positive peak (termed P) and from the baseline (0 µV) to the first negative trough (termed MeNR). In addition to the rod-isolated ERG, the PhNR and DA01 responses were also recorded in the same session. For PhNR measurement, the subject was exposed to a uniform blue field (12.5 cd/m^2^) for 2 min, and red flashes (3.0 cd-s/m^2^) were superimposed on the blue field to elicit the PhNR. A minimum of 5 responses were obtained and averaged. The PhNR amplitude was measured from the baseline (0 µV) to the trough of the PhNR (typically occurring at approximately 70 ms after the flash), consistent with ISCEV guidelines [1]. The DA01 responses were obtained according to ISCEV standards [19]. Specifically, the subject was dark-adapted for 20 min and an achromatic 0.01 cd-s/m^2^ flash was presented in the dark. A minimum of three responses were obtained and averaged. The DA01 amplitude was measured from the baseline (0 µV) to the peak of the b-wave, consistent with ISCEV recommendations [19].

## Results

**Fig. 1:**
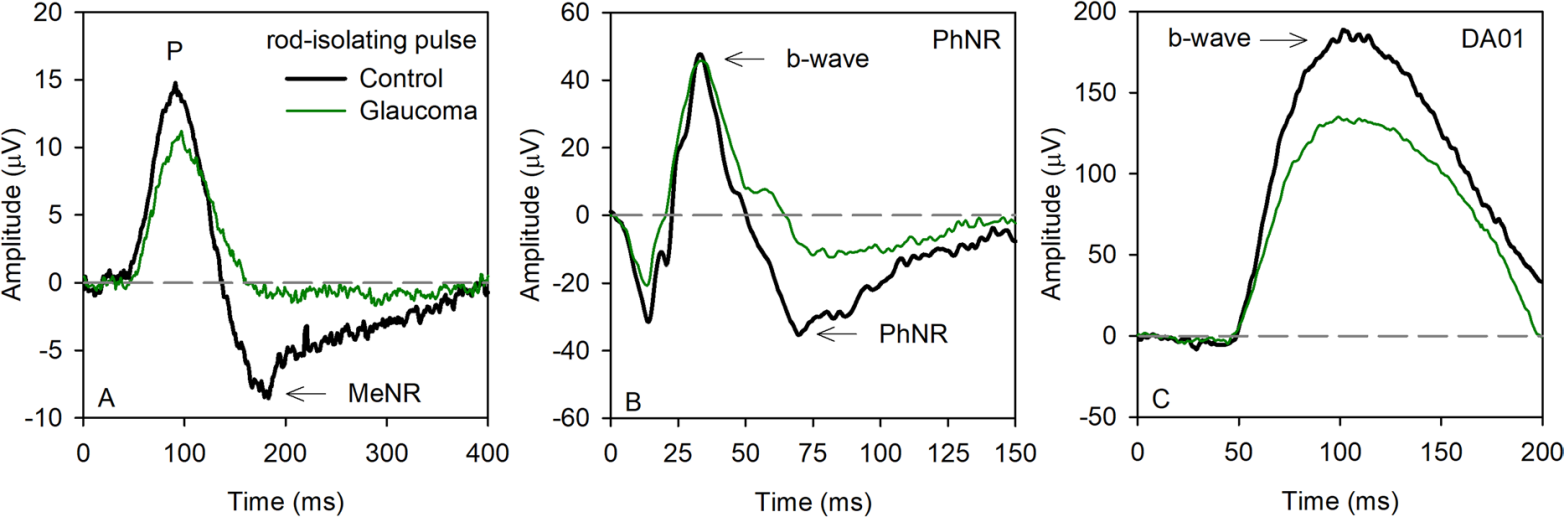
Mean waveforms for the control (heavy black) and glaucoma (thin green) subject groups. The mean rod-isolated response is shown in (**A**), the PhNR is shown in (**B**), and the ISCEV DA01 response is shown in (**C**)

Figure 1 shows mean waveforms for the control (black) and glaucoma (green) subject groups. The mean rod-isolated response is shown in (A), the PhNR is shown in (B), and the ISCEV DA01 response is shown in (C). For the glaucoma group, the rod-isolated positive potential (P) was slightly reduced, whereas the MeNR was nearly extinguished. Note the slow time-course of the onset (latency) of the P component, which occurs after the 40 ms rod-isolating pulse was extinguished. Panel B shows that the photopic a-wave was slightly reduced for the glaucoma group, the b-wave was also slightly reduced when measured from the trough of the a-wave to the b-wave peak, whereas the PhNR was substantially reduced. Panel C shows a modest reduction of the DA01 b-wave in the glaucoma group compared to the control group. These waveforms are presented to provide a superficial overview of the shape of the responses and the effects of severe glaucoma; components of these waveforms were extracted for quantitative analyses.

**Fig. 2:**
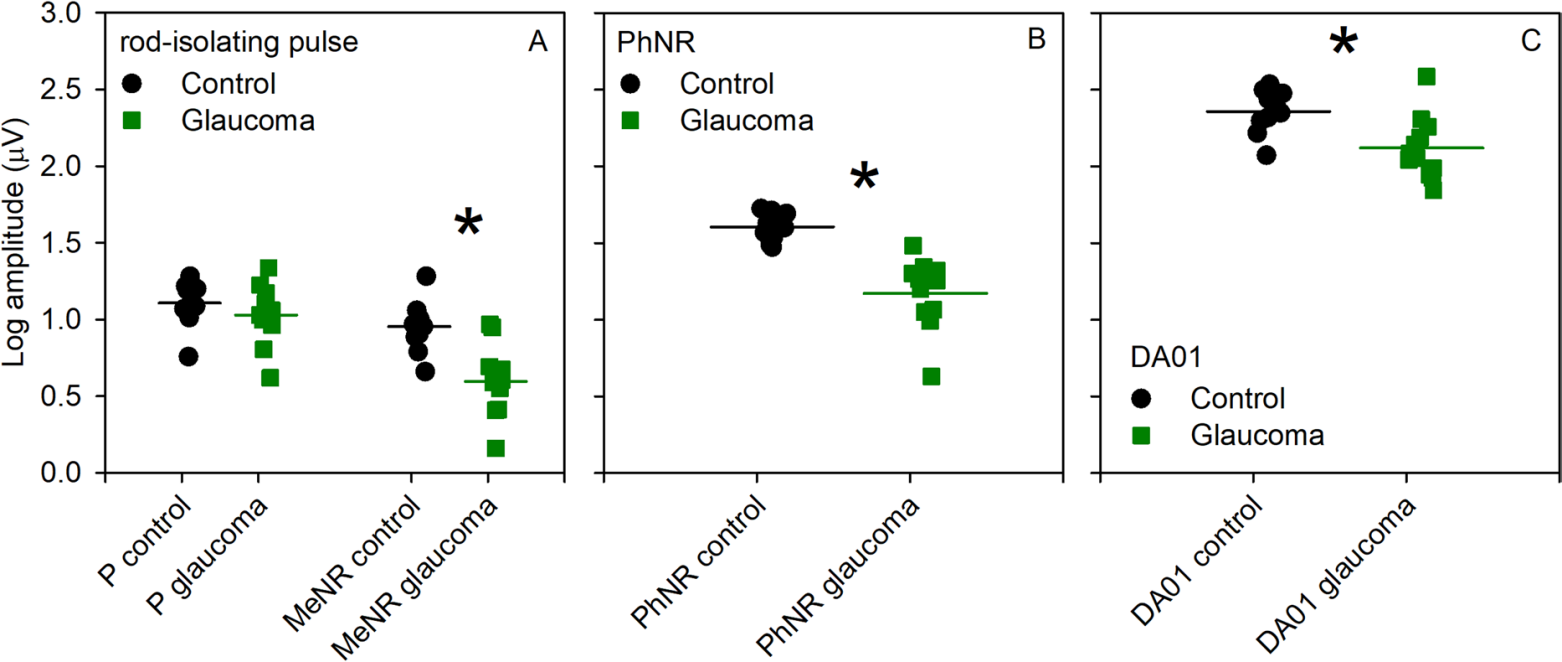
Log amplitude for each control (black circles) and glaucoma (green squares) subject; group averages are indicated by the horizontal bars. The left two data sets in panel **A** represent the amplitude of the positive (P) component of the rod-isolating pulse and the right two data sets in panel **A** represent the amplitude of the negative (MeNR) component. Panel **B** shows the PhNR amplitude, whereas the Panel **C** shows the DA01 b-wave amplitude. Asterisks mark statistically significant differences from the control group

Figure 2 plots the log amplitude for each control (black circles) and glaucoma (green squares) subject; group averages are indicated by the horizontal bars. The left two data sets in panel A represent the amplitude of the positive, P, of the rod-isolating pulse and the right two data sets in panel A represent the amplitude of the negativity, MeNR. The amplitude of P was similar for the control and glaucoma subjects, whereas the MeNR amplitude was attenuated for most of the glaucoma subjects. A repeated measures two-way analysis of variance (ANOVA), with main effects of group (control, glaucoma) and response component (P, MeNR) was performed to compare the amplitudes between the groups. There were significant effects of group (F = 12.80, *p* = 0.002) and component (F = 30.80, *p* < 0.001), as well as a significant interaction between these main effects (F = 6.88, *p* = 0.02). Holm-Sidak pairwise comparisons indicated no statistically significant amplitude difference in the P between the control and the glaucoma groups (t = 0.99, *p* = 0.33), but there was a significant amplitude difference for the MeNR (Fig. 2 asterisk; t = 4.42, *p* < 0.001). In addition, the log MeNR/P ratio was calculated for each subject (supplemental material); a t-test indicated a significant difference between the control and glaucoma groups in the log MeNR/P ratio (t = 2.69, *p* = 0.01). Panel B shows a reduction in PhNR amplitude for the glaucoma subjects, with most falling below the control range. A t-test indicated a significant difference between the control and glaucoma groups (asterisk; t = 5.59, *p* < 0.001). Panel C shows variability in the DA01 b-wave amplitude among the glaucoma subjects, but the mean amplitude was reduced and several individual subjects fell below the range of normal. A *t* test indicated a significant difference between the control and glaucoma groups in DA01 b-wave amplitude (asterisk; t = 3.21, *p* = 0.004). ROC analyses indicated that the MeNR and PhNR were both able to separate the control and glaucoma groups with high sensitivity and specificity, as expected given the advanced disease stage of the patient sample that was recruited (Supplemental material).

**Fig. 3:**
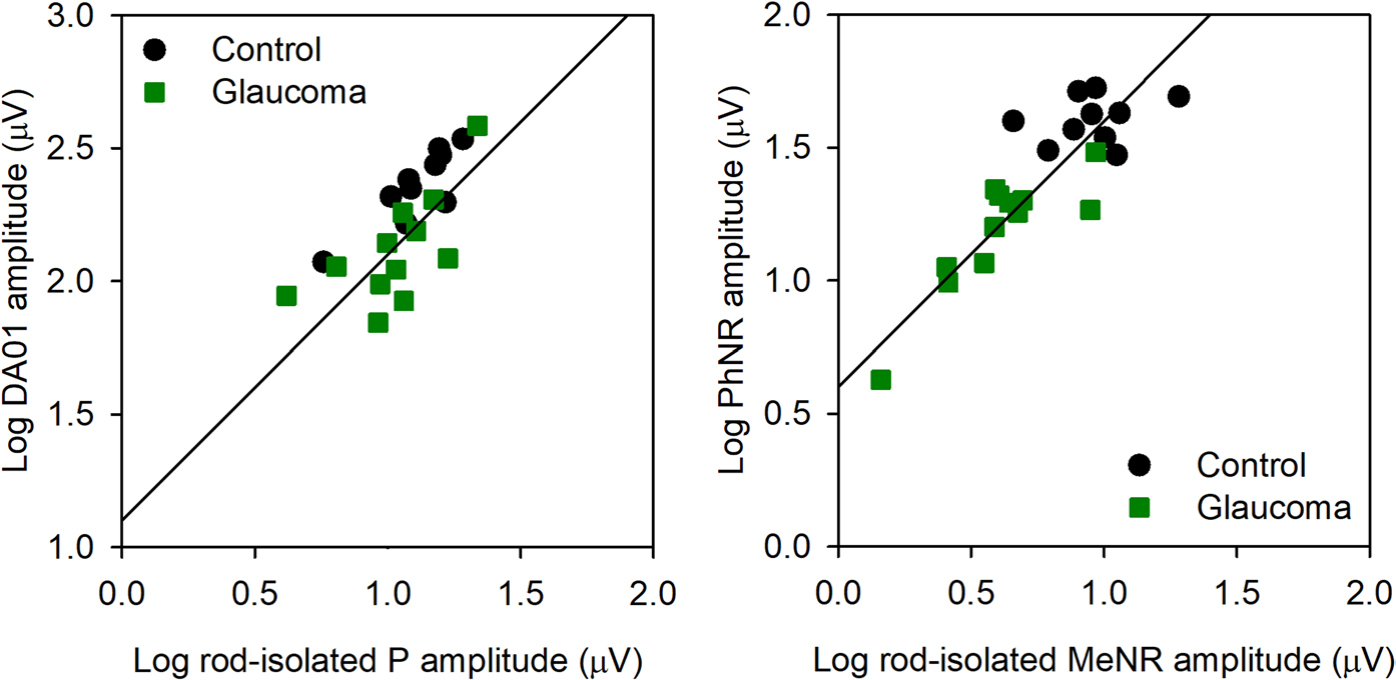
(left) Shows the correlation between log DA01 amplitude and log rod-isolated P amplitude for the control (black circles) and glaucoma (green squares) subjects. The right panel shows the correlation between log PhNR amplitude and log MeNR amplitude

Figure 3 (left) shows the correlation between DA01 log amplitude and rod-isolated P log amplitude for the control (black circles) and glaucoma (green squares) subjects. The solid line has a slope of 1.0 and is fit to the mean data. There were significant correlations between the DA01 and P amplitudes for the control group (r = 0.86, *p* = 0.001), glaucoma group (r = 0.67, *p* = 0.02), as well as for the two groups combined (r = 0.71, *p* < 0.001). The right panel shows the correlation between log PhNR amplitude and log rod-isolated MeNR amplitude. The solid line has a slope of 1.0 and is fit to the mean data. Although there was no significant correlation between the PhNR and MeNR amplitudes within the control group (r = 0.26, *p* = 0.46), the correlation between PhNR and MeNR was strong for the glaucoma group (r = 0.86, *p* < 0.001), as well as for the two groups combined (r = 0.86, *p* < 0.001).

Associations between the clinical parameters (HVF 24–2 MD and mean RNFL thickness) and the ERG parameters (rod-isolated P, MeNR, PhNR, photopic b-wave, and the DA01 amplitudes) were explored. HVF MD was not significantly correlated with any of the ERG parameters (all r < 0.36, *p* > 0.25). Likewise, the mean RNFL thickness was not significantly correlated with any of the ERG parameters (all r < 0.57, *p* > 0.05), with the exception of the rod-isolated P amplitude (r = 0.79, *p* = 0.002). Although not statistically significant, there were trends for thinned RNFLs to be associated with small DA01 (r = 0.57, *p* = 0.05) and photopic b-wave (0.48 *p* = 0.12) amplitudes.

## Discussion

The purpose of this study was to record rod-isolated ERGs elicited by short-duration (40 ms) pulses and to infer the generators of waveform components. The three primary findings were: (1) robust responses that appear to reflect activity within the rod-pathway can be recorded without dark-adaptation; (2) the P component of the rod-isolated response appears to be generated by rod ON bipolar cells, consistent with previous findings [12]; (3) the MeNR component of the rod-isolated response appears to be generated by RGCs within the rod pathway. Taken together, the results indicate that expedient analysis of bipolar cell and RGC function within the rod pathway can be achieved without dark adaptation. Although the primary purpose of this study was to report a novel technique for the objective assessment of rod-pathway RGC function, the data also clearly show that the MeNR and PhNR can be considerably attenuated in severe glaucoma. This is expected, as only patients with known advanced disease were selected for inclusion. Nevertheless, the MeNR and PhNR both successfully separated the control and patient groups with good sensitivity (≥ 90%) and specificity (≥ 83%). Future application of the technique to individuals with less advanced disease is of interest.

Previous studies have reported the morphology of the human rod ERG obtained by silent substitution using sinewave (2–16 Hz) [14–16] or square-wave (2 Hz) flicker [12]. The approach of the present study was largely based on that of Maguire et al. [12] who used 2 Hz square-wave flicker. The primary difference from this previous study is that a shorter duration pulse was used (40 ms). The shorter duration stimulus allows the offset of the pulse to occur prior to the onset of the ERG response, which is relatively slow for the rod pathway (approximately 50 ms for the rod-isolated ERG and DA01 b-wave). With this approach, ON and OFF response component separation is de-emphasized, akin to the typical DA3.0 and LA3.0 brief flash ERGs recorded under clinical conditions. Although the stimulus durations differed between the current study and that of Maguire et al. [12], the resulting rod-isolated waveforms shared similarities. Specifically, both studies report a positive potential at approximately 90–130 ms following the onset of the flash. Compelling evidence provided by Maguire et al. [12] indicates that the positive potential is generated by the rod ON bipolar cells. Our results showing a strong correlation between the rod-isolated P and the DA01 amplitudes support their conclusion. Following the P component, the rod-isolated ERG reported by Maguire et al. [12] showed a slow negative component while the stimulus remained in the “on” (increment) phase, but they did not comment on the origin of this potential. Figure 1A of the present report also shows a slow negative potential. The two negative potentials may not share identical sources, given the differences in the stimuli that generated them. Nevertheless, we show that the slow negative component is markedly reduced (or nearly extinguished) in patients with severe POAG. This finding suggests that the slow negative response, which we refer to as the MeNR, likely originates within rod-pathway RGCs under the stimulus conditions of the present study. The finding that the amplitudes of the MeNR and PhNR are highly correlated (Fig. 3) further supports this hypothesis.

The P component of the rod-isolated response was modestly attenuated in this sample of severe POAG subjects. Likewise, the ISCEV DA01 response was also modestly, but significantly, attenuated. Interestingly, the P component of the rod-isolated response was correlated significantly with mean RNFL thickness, whereas the correlation between the MeNR and mean RNFL thickness was not significant. Note that the absence, or near absence, of the MeNR in some of the glaucoma subjects may have created a floor effect that affected the correlation with RNFL thickness. However, patients with the thinnest mean RNFL values (less than 50 µm) had the most attenuated rod-isolated P and MeNR amplitudes, as well as reduced DA01 b-wave amplitudes. These findings suggest that bipolar cell function may be affected in late- or end-stage glaucoma and/or photoreceptor inputs into the bipolar cells may be abnormal. Although POAG is not typically associated with full-field ERG abnormalities under standard ISCEV conditions, there have been reports of full-field ERG reductions in end-stage glaucoma [20, 21], but ERG full-field abnormalities in glaucoma remain controversial [8, 22]. Individuals with severe POAG were recruited in the present study to ensure profound RGC damage, helping us to infer the primary generators of the waveform components. This approach can be expanded in future work to determine if the rod-isolated response is affected in earlier stages of glaucoma, as recently suggested in a study of mesopic contrast sensitivity [23].

A limitation of the present study is that the STR of the full-field ERG was not measured. As noted in the Introduction, the STR is rarely measured in human subjects due to the requirement of careful dark adaptation, low luminance stimulation, and relatively poor signal-to-noise [8], but this measure may have provided a better comparison to the MeNR, as compared to the PhNR. Despite challenges in recording the STR, it would be of interest to compare STR measurements to the MeNR in future work. Additionally, it would be of interest to determine how the PERG, a photopic measure of RGC function, compares with the MeNR in glaucoma and other optic neuropathies. An additional limitation is the complexity inherent in implementing silent substitution stimuli, which could limit broader adoption in clinical electrophysiology laboratories. However, manufacturers could incorporate a silent substitution stimulus, such as that described here, into their software suites. Adoption of a silent substitution guideline by ISCEV would help standardize the technique and may promote broader implementation.

In conclusion, the rod-isolated ERG appears to provide a measure of bipolar cell and RGC function within the rod pathway. Importantly, this response can be obtained relatively quickly without dark-adaptation. The rod-isolated ERG could be of particular value in studying possible inner-retina dysfunction in patients with cone dystrophies from whom measurable PERG and PhNR measurements cannot be obtained. The approach may also be of value in studying RGC dysfunction within the rod pathway in more common retinal diseases, including glaucoma and diabetic retinopathy.

## Supplementary Information

Below is the link to the electronic supplementary material.

Supplementary file 1 (DOCX 132 KB)

## Declarations

### Statement on the welfare of animals

No animal experiments were performed.
