# Supplementary material for: The mesopic negative response (MeNR): a novel approach to assess retinal ganglion cell function within the rod pathway
Source: Doc Ophthalmol. 2025 Jul 9;151(3):195–203. doi: 10.1007/s10633-025-10040-3 (PMC12568855; doi:10.1007/s10633-025-10040-3)
Supplement: Supplementary file 1 — (DOCX 132 KB) [file 10633_2025_10040_MOESM1_ESM.docx]

**Supplemental Figure 1:**





The supplemental figure shows the ratio of MeNR/P for the control (black circles) and glaucoma (green squares) subjects (A). As noted in the Results, the glaucoma group had a significant reduction in the MeNR/P ratio (t = 2.69, p = 0.01). Panel B show the results of an ROC analysis for the MeNR amplitude (red) and MeNR/P ratio (blue). Both metrics were able to separate the control and glaucoma groups with high sensitivity and specificity (see supplemental table). Panel C show the results of an ROC analysis for the PhNR amplitude (red) and PhNR/b-wave ratio (blue). Both metrics were able to separate the control and glaucoma groups with high sensitivity and specificity (see supplemental table).

**Supplemental Table: ROC analyses**

|  | MeNR log amplitude | MeNR/P ratio | PhNR log amplitude | PhNR/b-wave ratio |
| --- | --- | --- | --- | --- |
| Sensitivity | 0.90 | 0.70 | 1.00 | 0.90 |
| Specificity | 0.83 | 0.83 | 0.92 | 0.75 |
| AUC | 0.90 | 0.78 | 0.99 | 0.84 |
